# Exploring the Role of Sex and Sexual Experience in Predicting American Indian Adolescent Condom Use Intention Using Protection Motivation Theory

**DOI:** 10.3389/fpubh.2018.00318

**Published:** 2018-11-12

**Authors:** Rachel Strom Chambers, Summer Rosenstock, Angie Lee, Novalene Goklish, Francene Larzelere, Lauren Tingey

**Affiliations:** International Health, Bloomberg School of Public Health, Johns Hopkins, Baltimore, MD, United States

**Keywords:** american indian, youth, adolescent behavior, sexual health promotion, HIV prevention

## Abstract

**Introduction:** American Indian (AI) youth experience poor sexual health outcomes. Research indicates the Protection Motivation Theory (PMT) is a robust model for understanding how sexual risk and protective behaviors are associated with condom use intention (CUI). Studies indicate the constructs of the PMT which influence CUI vary by sex and sexual experience. This analysis explores associations between PMT constructs and CUI by sex and sexual experience among AI youth who participated in the Respecting the Circle of Life (RCL) trial, a sexual and reproductive health intervention.

**Methods:** We analyzed baseline data from the sample of 267 AIs, ages 13–19, who participated in the evaluation. We examined CUI and PMT construct scores by sex and sexual experience utilizing generalized estimated equations and multiple regression models to test which PMT constructs were associated with CUI across sex and sexual experience.

**Results:** Twenty-two percentage of participants were sexually experienced; 56.8% reported CUI at baseline. We found several differences in scores in PMT constructs by sex and sexual experience including self-efficacy, response efficacy, vulnerability, severity, and extrinsic rewards. We also found constructs varied that were associated with CUI varied across sex and sexual experience. No PMT constructs were associated with CUI among sexually experienced youth.

**Conclusion:** Results provide support for developing, selecting and delivering sexual health programs by sex and sexual experience in American Indian communities. Girls programs should focus on internal satisfaction and self-worth while boys should focus on negative impacts of not using condoms. Programs for youth who are not sexually active should focus on negative impacts of not using condoms. Programs for sexually inactive youth should work to change peer norms around condom use and improve knowledge about the efficacy of condom use.

## Introduction

Compared with the general U.S. youth population, American Indian (AI) youth experience poor sexual and reproductive health outcomes. In 2013, the birthrate for American Indian/Alaskan Native (AI/AN) youth ages 15–19 was 31.1 per 1,000 live births, while in that same year the national rate reached an historic low of 24.2/1,000 (1). It follows that AI/AN youth have one of the highest rates of repeat teen births (2). Sexually transmitted infection (STI) rates are also high among AI/AN youth; in 2015, AI/AN Chlamydia rates among 10–14 year olds and 15–19 year olds were 6.3 and 2.9 times that of Whites (3). In that same year, Gonorrhea cases were 7.4 and 4.2 times that of Whites among 10–14 year olds and 15–19 year olds (3). Although the actual number of diagnosed cases of HIV among all AI/ANs is low, it may be underestimated due to racial misclassification (4).

Some research indicates sexual risk behaviors including early sexual initiation, incorrect and inconsistent condom use, and multiple sex partners, are higher among AI/AN youth than youth of other races/ethnicities (5–7). Further, research with AI/AN youth has yielded the following conclusions: “AI/AN youth face intense pressure to engage in sex at young ages” (8) and “Inconsistent and low condom use represents an immediate risk factor for HIV infection” (9).

Quantitative and qualitative research substantiating incorrect and inconsistent condom use among AI/AN youth is alarming, given how effective condoms are in preventing STIs, HIV and pregnancy (6, 7, 9). Thus, sexual and reproductive health interventions targeting AI/AN youth should focus on teaching information and practicing skills to increase knowledge, intentions and behaviors that are requisite precursors to condom use (10). Several groups are developing sexual health interventions for AI/AN youth, though evaluation of such interventions is challenging. Often youth participants in efficacy trials have not initiated sexual intercourse and therefore, actual condom use may not be observed during data collection (11–16). Therefore, among young and/or sexually inexperienced AI/AN youth, measuring intervention impact on condom use intention is important, and may be the most meaningful and immediate antecedent to actual condom use (12, 17, 18). Past research with youth shows a strong association between intention and behavior and demonstrates the utility of measuring condom use intention as a proxy for actual condom use, particularly among sexually inexperienced participants (19–21).

The Protection Motivation Theory (PMT) has been applied to understand and predict a wide variety of behaviors, including sexual behaviors (22–26). PMT postulates that when a person is confronted with a health threat, such as HIV infection, two mediating cognitive pathways are evoked: threat appraisal and coping appraisal (27). The threat appraisal pathway balances the individual's assessment of their vulnerability to a threat (e.g., HIV infection) and severity of that threat with the perceived intrinsic (interpersonal) and extrinsic (intrapersonal) rewards of a maladaptive behavior (e.g., not using a condom). The coping appraisal pathway balances the effectiveness of an adaptive behavior (e.g., condom use) with the threat (e.g., HIV infection) and a person's ability to perform the adaptive behavior in the context of the perceived cost of doing so. Together the threat and coping appraisal pathways combine to form protection motivation, or the intention to engage in the adaptive or maladaptive behavior.

Studies conducted with youth employing the PMT indicate it is a robust theoretical model for understanding and altering risk behaviors for STIs and HIV (24, 28–36). The PMT has been shown to predict sexual risk behaviors including condom use intention and actual condom use across a variety of adolescent populations (23, 30, 31, 36, 37). Specifically, the coping appraisal pathway (particularly response efficacy) has predicted intention to initiate sex among Bahamian youth and condom use intention among South African youth (30, 36).

Previous research shows the predictive power of PMT constructs varies across sex and sexual experience (31, 37, 38). Among Vietnamese youth, both qualitative and quantitative differences have been found in PMT constructs by sex: females reported higher self-efficacy for abstinence, while males had higher response costs associated with condom use (38). Among South African youth, vulnerability and response efficacy were significantly correlated with condom use intention among males, while these same PMT constructs plus self-efficacy, were significantly correlated with condom use intention among females (31). Additionally, in a qualitative study conducted with AI youth, males reported high extrinsic rewards associated with sexual intention while girls reported high intrinsic rewards. Youth of both sexes in this study also reported that self-efficacy and response efficacy were associated with sexual intention (39).

Regarding sexually inexperienced vs. experienced youth, several differences have been studied. Among Dutch youth, sexually inexperienced youth reported lower perceived intrinsic rewards associated with sex, while sexually experienced youth had higher self-efficacy for condom use (40). Among sexually experienced Namibian youth, perceived intrinsic rewards were more likely to be associated with engaging in sex than they were among sexually inexperienced youth. Further, sexually inexperienced youth reported higher self-efficacy than those who were sexually experienced (37). Interpreted together, past research leaves room for new research to deepen understanding of the role sex and sexual experience play in the relationship between PMT constructs and condom use intention among AI/AN youth.

A randomized controlled trial evaluating Respecting the Circle of Life (RCL), an HIV/STI prevention program rooted in the Protection Motivation Theory, was conducted with AI youth ages 13–19 in 2011 and 2012 (14, 15). A previous analysis found the RCL program significantly impacted condom use intention among girls, younger youth, and sexually inexperienced youth. Further, that analysis also showed that at baseline, the PMT constructs of response efficacy and severity were associated with condom use intention, and that RCL significantly impacts response efficacy at follow-up (41). These findings illustrate the theoretical mechanism that RCL operates to impact condom use intention in this sample; that RCL only impacted condom use intention among specific sub-groups of youth is of interest. Building upon this previous analysis and the literature citing differences in the predictive power of PMT across sex and sexual experience, further exploration is warranted, especially given the dearth of similar analyses among AI/AN youth (31, 37–40).

In the current study, we explore how PMT constructs are associated with condom use intention across sex and sexual experience among AI youth who participated in the evaluation of RCL. Specifically, we examine how the relationship between condom use intention and PMT constructs differs across sex and sexual experience. Results will deepen our understanding of the theoretical constructs of a commonly used theory impact condom use intention among AI/AN youth across sex and sexual experience. Findings will provide direction for selecting and tailoring interventions for subgroups of AI/AN youth (24, 42).

## Materials and methods

### Participants

Participants were from an evaluation of Respecting the Circle of Life: Mind, Body, and Spirit (RCL), an adapted evidence-based intervention for HIV risk reduction, rooted in PMT (14, 15). A total of 267 self-identified AIs ages 13–19 were recruited and enrolled in the trial through local schools and at public events in the participating tribal community through non-probability sampling. Trained paraprofessionals from the community described the purpose, study design and enrollment criteria. To enroll, youth ages >18 completed written informed consent; for youth ages < 18 years old, parental permission and assent were obtained. All consenting documents were labeled with a unique participant ID number and stored in a locked cabinet at the local study site. The study was approved by relevant tribal, and University research review boards. All approval documents are kept electronically in a shared folder and in hard copy format at the study office. This manuscript was approved by the governing body of the participating tribal community.

### Measures

Participants completed the Youth Health Risk Behavior Inventory (YHRBI), a self-report tool measuring sociodemographic variables, psychosocial intentions and behaviors, as well as the seven PMT constructs. Feedback and input from the participating tribal community was incorporated to ensure the YHRBI was culturally and linguistically relevant. Confirmatory factor analysis (CFA) was used to examine YHRBI subscales; items were removed if they diminished the Cronbach's alpha value for a particular factor (15).

Condom use intention was measured by one question in which participants were asked if they would use a condom if they had sex in the next 6 months. Response options included: yes, maybe, don't know, probably not, and no. For the purposes of this analysis, we dichotomized condom use intention with yes being coded as 1 and all other response options coded as 0. Sexual experience was measured as a composite variable comprising lifetime experience of vaginal or anal sex. Vaginal sex was defined as, “When a boy puts his penis inside the girl's vagina” and anal sex as, “When a boy puts his penis in the butt of another person.” If participants answered no to both questions they were categorized as no lifetime sexual experience.

The seven PMT constructs (self-efficacy, response efficacy, response cost, intrinsic reward, extrinsic reward, severity, and vulnerability) were measured with 38 items on a five-point Likert scale (Please see Table 1 for a complete description of the questions measuring each PMT construct).

**Table 1:** Subscales for assessing protection motivation theory constructs.

**Subscale**	**Scoring**	**Cronbach's α**	**Items within subscale**
**COPING APPRAISAL PATHWAY**			
Self-efficacy (lower score = higher risk)	Range 1–5: 1 = Strongly agree 5 = Strongly disagree	0.67	I want to wait until I'm married before I have sex.^*^ If didn't want to have sex with someone going out with, I wouldn't be able to say no.^*^ If my sexual partner offers me drugs or alcohol I should take them. If my sexual partner uses drugs or alcohol before sex I should use them too.
Response efficacy (lower score = higher risk)	Range 1–5: 1 = Strongly disagree 5 = Strongly agree	0.68	If a girl says she won't have sex, a boy would say it's okay.^*^ Condoms are an important way to prevent pregnancy. Condoms are an important way to prevent you from getting a STD. Condoms are an important way to prevent you from getting HIV/AIDS.
Response cost (higher score = higher risk)	Range 1–5: 1 = Strongly disagree 5 = Strongly agree	0.61	My friends expect me to try drugs. My friends would think I was scared if I didn't try alcohol or drugs. If a girl carries condoms people think she is having sex. Condoms make sex hurt for a girl. Condoms make sex feel less good. When a guy and a girl are in a serious relationship they don't use condoms. Kids don't want other kids to think they are using condoms. Boys think it is important to have sex to feel like a man. Girls think it is important to have sex to feel like a woman.
**THREAT APPRAISAL PATHWAY**			
Intrinsic reward (higher score = higher risk)	Range 1–5: 1 = Very bad 5 = Very good Range 1–5: 1 = Strongly disagree 5 = Strongly agree	0.86	IF FOLLOWING HAPPENED IN THE NEXT 6 MONTHS, I WOULD FEEL…: Smoke marijuana (pot, grass, weed). Get an HIV infection. Drink alcohol (beer, whiskey, liquor, wine) Get an STD, (sexually transmitted disease, e.g., gonorrhea, herpes) Use cocaine Get pregnant or get a girl pregnant. Get suspended from school Have sex. I would like to know what it feels like to take drugs.
Extrinsic reward (higher score = higher risk)	Range 1–5: 1 = Strongly disagree 5 = Strongly agree Range 1-5: 1 = None 5 = Most	0.70	It is important that my friends respect me. Everyone my age has sex. My friends would lose respect for me if they thought I had an STD. How many of your close friends have sex. How many of the boys you know have sex? How many of the girls you know have sex?
Severity (higher score = higher risk)	Range 1–5: 1 = Strongly disagree 5 = Strongly agree	0.32	People who use drugs get HIV/AIDS. If two people are going together and one gets an STD, they would break up. If my mother knew I had an STD, she would be really upset.
Vulnerability (higher score = higher risk)	Range 1–5: 1 = No 2 = Probably not 3 = Don't know 4 = Maybe 5 = Yes	0.79	IN THE NEXT SIX MONTHS I WILL: Smoke marijuana (pot, grass, weed) (including just trying it once) Become infected with HIV. Drink alcohol, (beer, whiskey, liquor, wine) including just trying it once. Get an STD, (sexually transmitted disease, e.g., gonorrhea, herpes) Get pregnant/get a girl pregnant.

* *Values were re-coded in opposite direction*.

### Procedure

Participants completed the baseline assessment on hard-copy in a private room which took approximately 30–45 min. Research assistants supervised data collection to answer questions and address literacy issues, if needed. To ensure confidentiality, hard-copy data was entered into a secure database. All data were collected using a unique participant identification number with as few identifiers as possible. All hard copy data is stored in participant folders in a locked cabinet at the study site. Data is downloaded from the secure database and stored on a secure server that is HIPPA compliant. Our statistician has access to the data and is responsible for all statistical analyses.

### Analysis

Statistical analyses were carried out by the statistician on the study using Stata 14 statistical software (43). Bivariate analyses examining whether condom use intention and PMT constructs differed by sex and/or lifetime sexual experience were carried out using generalized estimating equations (GEE) with robust variance (see Tables 1, 2). Dichotomous outcomes used a Poisson family and log link, while continuous outcomes used a Gaussian family and identity link. The Poisson distribution was used because condom use intention was quite high (56.8%) and logistic regression with an outcome this common would likely over estimate Relative Risks (RR) (45). After baseline data was collected and prior to randomization, participants were asked to form sex and age-specific teams (15). Models were adjusted for self-selected peer-group clusters.

**Table 2:** Sex and sexual experience differences in protection motivation theoretical constructs relevant to condom use intention.

**Variable**	***n***	**Overall**	**Girls**	**Boys**	***p*-value**	**Inexperienced**	**Experienced**	***p*-value**
Condom use intention, %(*n*)	260	56.8%(148)	59.6%(87)	53.3%(61)	0.4127	49.4% (101)	81.2% (47)	< 0.0001
**COPING APPRAISAL, MEAN (SD)**								
Self-efficacy	261	4.28 (0.04)	4.47 (0.04)	4.04 (0.08)	< 0.0001	4.36 (0.06)	4.00 (0.09)	< 0.0001
Response-efficacy	261	3.77 (0.04)	3.68 (0.04)	3.89 (0.09)	0.0341	3.72 (0.06)	3.95 (0.06)	0.0081
Response-cost	262	2.86 (0.04)	2.87 (0.06)	2.85 (0.06)	0.8623	2.88 (0.04)	2.81 (0.08)	0.4273
**THREAT APPRAISAL, MEAN (SD)**								
Severity	262	3.65 (0.05)	3.77 (0.06)	3.49 (0.08)	0.0075	3.65 (0.05)	3.61 (0.10)	0.6902
Vulnerability	262	1.66 (0.06)	1.65 (0.07)	1.68 (0.11)	0.7930	1.56 (0.06)	1.99 (0.10)	< 0.0001
Extrinsic rewards	263	3.19 (0.06)	3.24 (0.08)	3.12 (0.08)	0.2793	3.00 (0.06)	3.79 (0.09)	< 0.0001
Intrinsic rewards	264	1.65 (0.06)	1.56 (0.08)	1.77 (0.10)	0.1050	1.60 (0.07)	1.80 (0.09)	0.0488

*All models have been adjusted for self-selected peer group clusters (44)*.

**Table 3:** Correlation between protection motivation theoretical constructs.

	**Self-efficacy**	**Response-efficacy**	**Response cost**	**Severity**	**Vulnerability**	**Extrinsic rewards**
Response-efficacy	0.2346^***^	–	–	–	–	–
Response cost	−0.1205^*^	−0.0212	–	–	–	–
Severity	0.2735^***^	0.1762^**^	0.074	–	–	–
Vulnerability	−0.4398^***^	−0.0949	0.1916^**^	−0.1161	–	–
Extrinsic rewards	−0.0388	0.1300^*^	0.1353^*^	0.0841	0.1738^**^	–
Intrinsic rewards	−0.4599^***^	−0.0705	0.1016	−0.0831	0.4863^***^	0.0900

* *p < 0.05*,

** *p < 0.01*,

*** *p < 0.005*.

Pairwise Pearson's correlation coefficients were calculated to determine the strength and statistical significance of the correlations between the PMT constructs in this study population. Finally, four separate multiple regression models were built to test which PMT constructs in context were associated with condom use intention among: (1) girls, (2) boys, (3) sexually experienced participants, and (4) sexually inexperienced participants. GEE with a Poisson family, log link and robust variance, adjusted for self-selected peer group clusters was used to build these models. Tables 4, 5 present fully saturated and the final, more parsimonious explanatory models, for each category. The final models were produced to better understand which of the PMT constructs in context were most strongly associated with condom use intention. The final models retained some variables that were marginally statistically significant. Although these variables' *p*-values are equal to or slightly above 0.05, the RRs remain meaningful and inclusion of the variables is important to the stability of the model as there is some evidence of mild multi-collinearity. Some level of multi-collinearity among these constructs was expected as they are all domains within a larger theoretical framework. All results of data analysis were reviewed by the study team, including the principle investigator, the study manager, the statistician and local Native study staff to ensure accurate interpretation. Additionally, members of the governing body of the Tribal community (made up of elected members of the tribal community) were given copies of the results to review. The results and interpretation of the results were presented by study staff at a meeting with the governing body. Written feedback was gathered by the local study staff during this meeting and discussed with the principle investigator, program manager and statistician. The written feedback is stored in a word document in a shared study folder.

**Table 4:** Association between condom use intention and Protection Motivation Constructs by sex.

**PMT Constructs**	**Girls**		**Boys**	
	**Full model (*N* = 144)**	**Final model (*N* = 144)**	**Full model (*N* = 113)**	**Final model (*N* = 113)**
	**RR (95% CI)**	**RR (95% CI)**	**RR (95% CI)**	**RR (95% CI)**
Self-efficacy	1.07 (0.75–1.52)	–	1.15 (0.79–1.67)	–
Response efficacy	1.15 (0.99–1.32)	1.16 (0.99, 1.37)	1.45 (1.10–1.91)	1.55 (1.17, 2.07)
Response cost	0.83 (0.68–1.02)	–	0.81 (0.57–1.14)	–
Severity	1.12 (0.93–1.35)	–	1.19 (0.96–1.49)	1.22 (1.00, 1.48)
Vulnerability	0.88 (0.71–1.11)	0.84 (0.71, 0.99)	1.44 (0.98–2.13)	1.22 (1.02, 1.45)
Extrinsic rewards	1.22 (1.02–1.47)	1.21 (0.99, 1.47)	1.32 (1.02–1.71)	1.24 (0.99, 1.55)
Intrinsic rewards	1.17 (1.08–1.27)	1.20 (1.07, 1.34)	0.90 (0.70–1.17)	–

*All models have been adjusted for self-selected peer group clusters (44)*.

**Table 5:** Association between condom use intention and Protection Motivation Constructs by sexual experience.

**PMT Constructs**	**Sexually experienced**		**Sexually inexperienced**	
	**Full model (*N* = 59)**	**Final model (*N* = NA)**	**Full model (*N* = 198)**	**Final model (*N* = 198)**
	**RR (95% CI)**	**RR (95% CI)**	**RR (95% CI)**	**RR (95% CI)**
Self-efficacy	0.84 (0.67–1.06)	–	1.27 (0.89–1.81)	–
Response efficacy	1.16 (0.98–1.38)	–	1.22 (0.99–1.49)	1.27 (1.01, 1.59)
Response cost	0.88 (0.72–1.08)	–	0.89 (0.67–1.16)	–
Severity	1.03 (0.84–1.28)	–	1.20 (0.96–1.51)	1.28 (1.05, 1.56)
Vulnerability	1.10 (0.88–1.38)	–	1.03 (0.82–1.29)	–
Extrinsic rewards	0.84 (0.66–1.07)	–	1.26 (1.03–1.53)	1.24 (1.02, 1.52)
Intrinsic rewards	0.77 (0.55–1.08)	–	1.08 (0.92–1.26)	–

*All models have been adjusted for self-selected peer group clusters (44)*.

## Results

### Participant characteristics (data not shown in tables)

Participants were 267 AI youth ages 13 to 19 (mean age = 15.13 years; *SD* = 1.65) and 56% (*n* = 150) were girls. Most participants were in school (93.3%) and 29.8% had been suspended. Twenty-two percent (*n* = 59) reported lifetime sexual experience at baseline and 17.7% (*n* = 47) reported vaginal or anal intercourse in the past 6 months. More boys reported lifetime sexual experience than girls (29.1 vs. 16.8 %; *p* = 0.017). Of those sexually experienced, 78.2% (*n* = 43) reported using a condom at last sex, which was not significantly different between boys and girls.

### PMT constructs and condom use intention across sex and sexual experience (tables 2, 3)

Overall, 56.8% of participants had condom use intention at baseline. Condom use intention did not differ significantly by sex, but did differ significantly between sexually experienced and inexperienced youth (81.2 vs. 49.4%, *p* < 0.0001). Mean scores on PMT variables varied across sex and sexual experience. Specifically, girls reported higher self-efficacy to avoid sexual risk behaviors (4.47 vs. 4.04; *p* < 0.0001) and higher perceived severity associated with the consequences of sexual risk behaviors (3.77 vs. 3.49; *p* = 0.0075) than boys. Boys had higher response efficacy scores (3.89 vs. 3.68; *p* = 0.0341), or the belief that condoms are efficacious in preventing HIV/STIs and pregnancy. Sexually experienced youth had higher response efficacy than inexperienced youth (3.95 vs. 3.72; *p* = 0.0081) (belief that condoms are efficacious); they were also more likely to feel vulnerable to infection and unintended pregnancy (1.99 vs. 1.56; *p* ≤ 0.0001). Sexually experienced youth reported feeling at higher risk for engaging in sexual risk behaviors due to extrinsic (3.79 vs. 3.00; *p* < 0.0001) and intrinsic rewards (1.60 vs. 1.89; *p* = 0.0488). Sexually inexperienced youth reported higher self-efficacy to avoid sexual risk behaviors than experienced youth (4.36 vs. 4.00; *p* < 0.0001). Self-efficacy was moderately correlated with vulnerability and intrinsic rewards, and weakly correlated with all other variables except extrinsic rewards. Vulnerability was also moderately correlated with intrinsic rewards. Further, many of the constructs are correlated with more than one other construct. We expected this multi-collinearity as they are constructs of a comprehensive theoretical model.

### Associations with condom use intention across sex (table 4)

Among girls, extrinsic rewards (RR = 1.22; CI = 1.02 = 1.47) and intrinsic rewards (RR = 1.17; CI = 1.08–1.27) predicted condom use intention in the fully saturated model. These two variables as well as response efficacy (RR = 1.16; CI = 0.99–1.37) predicted condom use intention in the final multivariate model; while vulnerability reduced the likelihood of having condom use intention (RR = 0.84; CI = 0.71–0.99). Among boys, response efficacy (RR = 1.45; CI = 1.10–1.91) and extrinsic rewards (RR = 1.32; CI = 1.02–1.71) predicted condom use intention in the fully saturated model. In the final multivariate model, response efficacy (RR = 1.55; CI = 1.17–2.07), severity (RR = 1.22; CI = 1.00–1.48), vulnerability (RR = 1.22; CI = 1.02–1.45), and extrinsic rewards (RR = 1.24; CI = 0.99–1.55) predicted condom use intention. Among both girls and boys, additional variables were retained in the final model due to the presence of moderate multi-collinearity (see section Materials and Methods).

### Associations with condom use intention across sexual experience (table 5)

Among sexually inexperienced youth, extrinsic rewards were associated with condom use intention in the fully saturated model (RR = 1.26; CI = 1.03–1.53) while response efficacy (RR = 1.22; CI = 0.99–1.49) and severity (RR = 1.20; CI = 0.96–1.51) were marginally significant. In the final model for sexually inexperienced participants, response efficacy (RR = 1.27; CI = 1.01–1.59), severity (RR = 1.28; CI = 1.05–1.56), and extrinsic rewards (RR = 1.24; CI = 1.02–1.52) were associated with condom use intention. Among sexually experienced youth, no PMT variables were associated with condom use intention in the fully saturated or final models.

## Discussion

### Summary of findings

This is the first study to examine condom use intention across sex and sexual experience through the lens of Protection Motivation Theory among a sample of AI youth, and builds on previous analysis conducted by Tingey et al. (41). Results indicate PMT constructs associated with condom use intention vary across sex and sexual experience and no PMT constructs are associated with condom use intention among sexually active Native youth in this sample.

Among boys, girls and sexually inexperienced sub-groups in this study, at least two of the four threat appraisal pathway constructs of the PMT (intrinsic and extrinsic rewards, vulnerability and severity) were associated with condom use intention. The role of the threat appraisal pathway in predicting condom use intention across these sub-groups in this population is of interest as many studies have found the coping appraisal pathway (particularly self-efficacy) to be a stronger predictor of outcomes of interest (30, 36). Additionally, the PMT self-efficacy construct did not appear to be associated with condom use intention among our sub-groups in this analysis. In a previous analysis of this data, condom use self-efficacy was found to be associated with condom use intention at baseline (41). Future research should explore the relationship between condom use intention and global vs. action-specific efficacy (i.e., self-efficacy vs. condom use self-efficacy) given self-efficacy is an established predictor of risk behaviors and a target in many sexual risk reduction interventions (29, 31, 36, 37, 46).

### Limitations

While there are many strengths of this study, there are limitations. First, this analysis assessed factors of motivation looking only at the PMT, which does not take into account environmental, structural or historical factors that my influence knowledge, intentions, beliefs or experiences related to condom use intention. The goal of this paper was to further understand the theoretical framework that underpins the RCL program, one of the only programs adapted for and implemented with AI youth. Second, because this is cross sectional data, we cannot make conclusions about causality. Third, we only measured intention to use a condom and not actual condom use. While there is a strong association between these two measures, we were unable to assess the relationship between PMT constructs and actual condom use due to the small sample size of sexually active youth in the original trial. However, regarding HIV/AIDS prevention, multiple evaluations of behavioral interventions indicate a positive predictive relationship between condom use intention and actual condom use (19–21). Third, the primary outcome, condom use intention was assessed via a single item dichotomous variable. Additionally, PMT constructs were measured using an assessment previously developed and utilized by Stanton et al. for African American youth (47). While the YHRBI was not developed with or for AI populations, CFA was used to retain individual scale items that most consistently measured the hypothesized constructs (15). The severity construct alpha was low. Therefore, it should be interpreted with caution and future use of this scale should consider potentially adapting the questions associated with severity. Finally, no constructs were found to be significant among sexually active youth. The number of youth in this sample who were sexually active is small and over 80% of them had condom use intention, leaving < 11 sexually active youth who did not have condom use intention. With this small of a sample, finding a difference between groups with and without condom use intention would be very difficult to identify. Thus, with a larger sample size of sexually active youth, there is a possibility one would find associations between PMT constructs and CUI.

### Implications

This study has improved our understanding of the associations between condom use intention and PMT constructs among AI youth across sex and sexual experience. Our findings have several implications for the development of sexual risk reduction programs targeting AI youth. Based on the difference in the role of PMT constructs across sex and sexual experience, HIV prevention education and sexual risk reduction efforts need to target different PMT constructs for girls and boys. Importantly, the lack of association of PMT constructs among sexually active youth indicates that programs based in PMT may not be appropriate for this group. Programs based in other behavioral change models such as the theory of reasoned action and theory of planned behavior should be considered among sexually active Native youth. Constructs of both of these theories have been shown to influence condom use intention in other samples of sexually active youth (19). Potentially, it may be advantageous to screen youth for sexual activity status prior to enrolling them in sexual health programs so as to help identify the most efficacious program for them based on their gender and sexual experience.

Delivery to peer groups organized by sex and age-groups, may be advantageous as it would allow tailored messaging and programming for these sub-groups of youth. Traditional sexual health education programs, including those in schools, must often teach uniform curriculum to classrooms of mixed sexes and sexual experience. In AI communities, alternative community-based methods for program delivery, such as through summer camps, may provide greater flexibility, and allow grouping youth by sex and sexual experience. This allows for tailoring curriculum content and/or selection of different curricula for different groups, thereby resulting in higher impact. That extrinsic rewards, the perception that peers are participating in risky behaviors, predicted condom use intention across sex and sexual experience provides additional evidence that delivering programs to peer groups may be advantageous (15, 48). Additionally, programs that work to change peer norms, discuss values, and include strategies to cope with peer pressure, such as Respecting the Circle of Life, may wield greater influence than those not targeting these extrinsic rewards (15, 49).

We found across both males and females as well as sexually inexperienced youth that response efficacy predicts condom use intention. This is consistent with other studies assessing the impact of PMT components on sexual risk behaviors (36, 41). Thus, programs targeting youth should strive to build response efficacy through discussions, demonstrations, and role playing of the use of condoms for STI/HIV and pregnancy prevention. In previous qualitative research conducted with AI youth, participants discussed lack of knowledge about birth control, including condoms; they didn't understand how different birth control methods were used or their efficacy (39). Based on the results presented in this paper and by Chambers et al. including information to increase youth knowledge about different forms of birth control should be included in sexual health promotion programs (39). Promoting partner negotiation skills among youth may help improve youth's belief that condoms and talking to their partner work in preventing STIs and teen pregnancy (50, 51).

The role of internal satisfaction (intrinsic rewards) in condom use intention needs to be explored. It is clear that youth who obtain internal satisfaction from sexual risk behaviors are less likely to use condoms and therefore, as a public health community, we need to seek ways to promote internal satisfaction through healthy behaviors as opposed to risk behaviors, especially among Native girls. The relationship between vulnerability and condom use intention should be interpreted with caution. When answering questions pertaining to vulnerability, youth may have taken into account their previous experience and/or intention to participate in risk behaviors. Still, the low scores and lack of a positive association between condom use intention and vulnerability among girls and youth who are not sexually active, is of interest. In a separate qualitative study assessing HIV risk behaviors and intentions among AI youth, participants reported low vulnerability to STIs and HIV infection. The authors concluded that low vulnerability may be tied to the lack of knowledge about certain risks factors for HIV/STIs resulting in an inability to recognize their own personal risk (39). This conclusion is further supported by a study conducted by Boer et al. which found inaccurate beliefs about HIV transmission lowered feelings of vulnerability (52). Therefore, activities that provide accurate information about risk behaviors and present the prevalence of HIV and STIs in similar communities should be included in HIV prevention programs. Providing accurate information about the consequences of risk behaviors among Native youth, especially inexperienced Native youth, may be especially impactful for moving the needle on condom use intention.

Findings from this study provide specific direction for the selection of and tailoring of sexual and reproductive health programs for different groups of AI/AN youth. Overall, this study provides rationale for the development and delivery of HIV prevention programs by sex and sexual experience. It underscores the importance of providing HIV prevention programs routed in PMT to youth before they become sexually active as constructs of the PMT are related to condom use intention only among youth who are sexually inexperienced. For sexually active youth, results indicate other programs that are not routed in PMT constructs may be more efficacious in improving condom use intention.

## Ethics statement

This study was carried out in accordance with the recommendations of the National Institutes of Health. The protocol was approved by the Johns Hopkins University IRB as well as the Phoenix area Indian Health Board Institutional Review Board. All subjects gave written informed consent in accordance with the Declaration of Helsinki.

## Author contributions

All authors contributed to the concept of the manuscript and reviewed and edited multiple drafts of the manuscript. RC drafted the manuscript, SR completed all data analysis and contributed to the methods and results section and LT drafted sections of the methods section.

### Conflict of interest statement

The authors declare that the research was conducted in the absence of any commercial or financial relationships that could be construed as a potential conflict of interest.
